# The Biology of COP Cells: Mesenchymal of Hematopoietic?

**DOI:** 10.1093/geroni/igab046.170

**Published:** 2021-12-17

**Authors:** Meghan McGee-Lawrence

**Affiliations:** Medical College of Georgia, Augusta University, Augusta, Georgia, United States

## Abstract

Circulating osteogenic precursor (COP) cells constitute a recently discovered population of circulating progenitor cells with the capacity to form not only bone but other mesenchymal tissues. A small but growing body of literature explores these cells, but with a great deal of disagreement and contradiction within it, mainly whether these cells are from mesenchymal or hematopoietic origin. This session will discuss the origins and biological characterization of these cells, including the identification strategies used to isolate these cells from the peripheral blood. It also examines the available knowledge on the in vitro and in vivo behaviour of these cells in plastic adherence, differentiation capacity, proliferation, and cellular homing. We will also review the profound and exciting implications for future use of COP cells in clinical practice, particularly in comparison with other types of stem cells.

